# Mortality of patients with hospital-onset sepsis in hospitals with all-day and non-all-day rapid response teams: a prospective nationwide multicenter cohort study

**DOI:** 10.1186/s13054-022-04149-z

**Published:** 2022-09-16

**Authors:** Dong-gon Hyun, Su Yeon Lee, Jee Hwan Ahn, Jin Won Huh, Sang-Bum Hong, Younsuck Koh, Chae-Man Lim, the Korean Sepsis Alliance (KSA) Investigators, Dong Kyu Oh, Gee Young Suh, Kyeongman Jeon, Ryoung-Eun Ko, Young-Jae Cho, Yeon Joo Lee, Sung Yoon Lim, Sunghoon Park, Jeongwon Heo, Jae-myeong Lee, Kyung Chan Kim, Yeon Joo Lee, Youjin Chang, Kyeongman Jeon, Sang-Min Lee, Suk-Kyung Hong, Woo Hyun Cho, Sang Hyun Kwak, Heung Bum Lee, Jong-Joon Ahn, Gil Myeong Seong, Song-I Lee, Sunghoon Park, Tai Sun Park, Su Hwan Lee, Eun Young Choi, Jae Young Moon

**Affiliations:** Department of Pulmonary and Critical Care Medicine, Asan Medical Center, University of Ulsan College of Medicine, 88 Olympic-ro 43-gil, Songpa-gu, Seoul, 05505 Republic of Korea

**Keywords:** Hospitals, hospital rapid response team, Sepsis, General ward, Hospital mortality, Lactic acid

## Abstract

**Background:**

Hospital-onset sepsis is associated with a higher in-hospital mortality rate than community-onset sepsis. Many hospitals have implemented rapid response teams (RRTs) for early detection and timely management of at-risk hospitalized patients. However, the effectiveness of an all-day RRT over a non-all-day RRT in reducing the risk of in-hospital mortality in patient with hospital-onset sepsis is unclear. We aimed to determine the effect of the RRT’s operating hours on in-hospital mortality in inpatient patients with sepsis.

**Methods:**

We conducted a nationwide cohort study of adult patients with hospital-onset sepsis prospectively collected from the Korean Sepsis Alliance (KSA) Database from 16 tertiary referral or university-affiliated hospitals in South Korea between September of 2019 and February of 2020. RRT was implemented in 11 hospitals, of which 5 (45.5%) operated 24-h RRT (all-day RRT) and the remaining 6 (54.5%) had part-day RRT (non-all-day RRT). The primary outcome was in-hospital mortality between the two groups.

**Results:**

Of the 405 patients with hospital-onset sepsis, 206 (50.9%) were admitted to hospitals operating all-day RRT, whereas 199 (49.1%) were hospitalized in hospitals with non-all-day RRT. A total of 73 of the 206 patients in the all-day group (35.4%) and 85 of the 199 patients in the non-all-day group (42.7%) died in the hospital (*P* = 0.133). After adjustments for co-variables, the implementation of all-day RRT was associated with a significant reduction in in-hospital mortality (adjusted odds ratio 0.57; 95% confidence interval 0.35–0.93; *P* = 0.024).

**Conclusions:**

In comparison with non-all-day RRTs, the availability of all-day RRTs was associated with reduced in-hospital mortality among patients with hospital-onset sepsis.

**Supplementary Information:**

The online version contains supplementary material available at 10.1186/s13054-022-04149-z.

## Introduction

Sepsis is a major health concern that causes significant mortality worldwide [1]. The International Surviving Sepsis Campaign recommends early identification and management of patients with sepsis and septic shock [2]. Hospital-onset sepsis (HOS) is when signs and symptoms of sepsis develop after hospital admission [3]. HOS accounts for approximately 10%-20% of sepsis cases and is associated with a higher in-hospital mortality rate than community-onset sepsis [4, 5]. In a study based on a national administrative database, patients with HOS admitted to the intensive care unit (ICU) showed a poorer prognosis and required more organ support than those with community-onset sepsis [6]. Nevertheless, patients with HOS are less likely to receive proper management than those with community-onset sepsis. The overall adherence to the sepsis bundle for patients with HOS was lower than that for patients with community-onset sepsis [7, 8]. Several obstacles, including the complicated clinical status of in-hospital patients, intermittently performed screening, and low comprehension of providers for the sepsis bundle, may be responsible for the reduced adherence to the sepsis bundle components in patients with HOS [7–9].

Over the past two decades, RRT has been implemented in many secondary or tertiary hospitals in the belief that early detection and timely management of at-risk patients will improve hospital outcomes [10–12]. Several recent studies have demonstrated this belief by showing that RRT could help reduce in-hospital mortality rates [13–18]. In addition, RRT implementation has been shown to improve the adherence to the sepsis bundle in HOS [9]. Moreover, in 2018, the Korean Health Insurance Review and Assessment Service and the Ministry of Health and Welfare distributed the third edition of revised standards that recommend all acute care hospitals implement an RRT. Afterward, more than 15 hospitals have implemented the RRT in Korea [19].

Nevertheless, RRT practice is not uniform across institutions and depends on the personnel composition, available resources, and regional hospital culture [20]. A single-center study reported that RRT operating hours are associated with a reduction in cardiopulmonary arrest [21]. However, the effects of differences in RRT practices on the prognosis of patients with HOS remain unclear. Therefore, this study aimed to determine the effect of RRT operating hours on the primary outcome of in-hospital mortality and the secondary outcome of adherence to sepsis bundle within 3 h in patients with HOS.

## Methods

### Study design and data sources

We conducted a secondary analysis of prospectively collected data from the Korean Sepsis Alliance (KSA) database between September of 2019 and February of 2020. All data used in this study were derived from the KSA database, which is a nationwide prospective registry for sepsis from 16 tertiary or university-affiliated hospitals in South Korea including 11 centers operating RRT [22]. The 11 hospitals with RRT consisted of 5 (45.5%) which operated for 24 h a day, three (27.3%) which operated for over 8 h a day, and three (27.3%) which operated for 8 h or less a day (Additional file 1: Table S1 & Table S2).

All of the hospitals were required to report data for patients with sepsis using electronic case report forms that included demographic characteristics, comorbidities, illness severity, sepsis characteristics, and clinical outcomes by trained coordinators reviewing each patient’s electronic medical records. These forms were stored in the KSA database. Timestamps for the initiation of bundled care were also required for patients who received sepsis treatment. Sepsis was defined as suspected infection with organ dysfunction based on the criteria suggested in the third international consensus definition for sepsis and septic shock (Sepsis-3) [23]. Quality assurance initiatives to ensure data completeness and accuracy were conducted regularly.

### Patient selection

We included all patients older than 19 years of age who had sepsis. We divided sepsis patients into two groups according to the location of occurrence. HOS was an occurrence of sepsis after patient arrival in the general ward, and community-onset sepsis was defined as a sepsis occurrence after patient arrival in the emergency department. For example, a patient who showed signs of infection in the emergency department but meets the criteria of sepsis after arriving in the general ward was categorized as having hospital-onset sepsis. To focus on patients with HOS, we excluded patients with community-onset sepsis.

### Variables

We performed bivariate analyses of the characteristics and hospital outcomes of patients in the hospitals that operated RRT 24-h a day (all-day group) and those in hospitals that did not operate an all-day RRT (non-all-day group). The primary outcome was to compare in-hospital mortality between the all-day group and the non-all-day group. We also evaluated other secondary outcomes, such as hospital length of stay, transfer to ICU, ICU mortality, ICU length of stay, treatments during ICU, and adherence to the three-hour bundle.

Covariates considered as potential confounders of hospital outcomes based on previous studies were extracted from the KSA database [8, 24, 25]. These variables included demographic factors such as age, sex, and body mass index (BMI), comorbidities, suspected infection site, blood culture positivity, including multidrug-resistant (MDR) pathogens, and measures of illness severity, such as the presence of septic shock, serum lactate level, or the sequential organ failure assessment score (SOFA). Baseline comorbidity was represented by a count of conditions from the Charlson comorbidity index (CCI) [26]. Septic shock was defined as a serum lactate level of at least 2 mmol/L and requiring vasopressors to maintain a mean arterial pressure of more than 65 mmHg despite adequate fluid resuscitation. The SOFA was calculated to evaluate the illness severity at the time of RRT activation for each patient [27]. If patients did not have pre-existing organ dysfunction, the baseline SOFA score was assumed to be zero.

We also collected data on the adherence to and time spent on the 3-h sepsis bundle, including blood culture results, broad-spectrum intravenous antibiotics treatment data, serum lactate level test results, and intravenous fluid treatment data if the blood pressure was low or the lactate level was elevated, along with data for initiation of vasopressor treatment, if indicated [28]. Time zero of sepsis was considered to be the time when RRT recognized patients with sepsis for the first time. The time to completion of the three-hour bundle was defined as the time in hours from time zero until all of the elements of the three-hour bundle were performed. Also, the time to completion of each element was measured from time zero until the completed element administration. If any element was performed before time zero, it was regarded as adherence when it was completed within the previous 48 h.

### Statistical analysis

Data were presented as numbers and proportions for categorical variables and means ± standard deviations or medians (interquartile range [IQR]) for continuous variables. Differences between groups were analyzed using the *χ*^2^ test or Fisher’s exact test and independent two-sample t-test or Mann–Whitney *U* test with a normal or non-normal distribution, as appropriate. Kaplan–Meier analysis was used to estimate the cumulative incidence of adherence to the completion of the three-hour bundle, whereas a log-rank test was used to test the significance of the differences. Multivariable modeling of the association between the RRT operating time and in-hospital mortality was performed via logistic regression, with adjustments for covariables. We selected covariables with *P* values < 0.05 in the univariable analysis. A final model was constructed using independent covariables, considering multicollinearity based on associations in the scientific literature [22, 23]. Two-sided *P* values < 0.05 indicated significance. All analyses were performed using SPSS ver. 24.0 (IBM Corporation, Armonk, NY) software.

## Results

### Patient selection

**Fig. 1:**
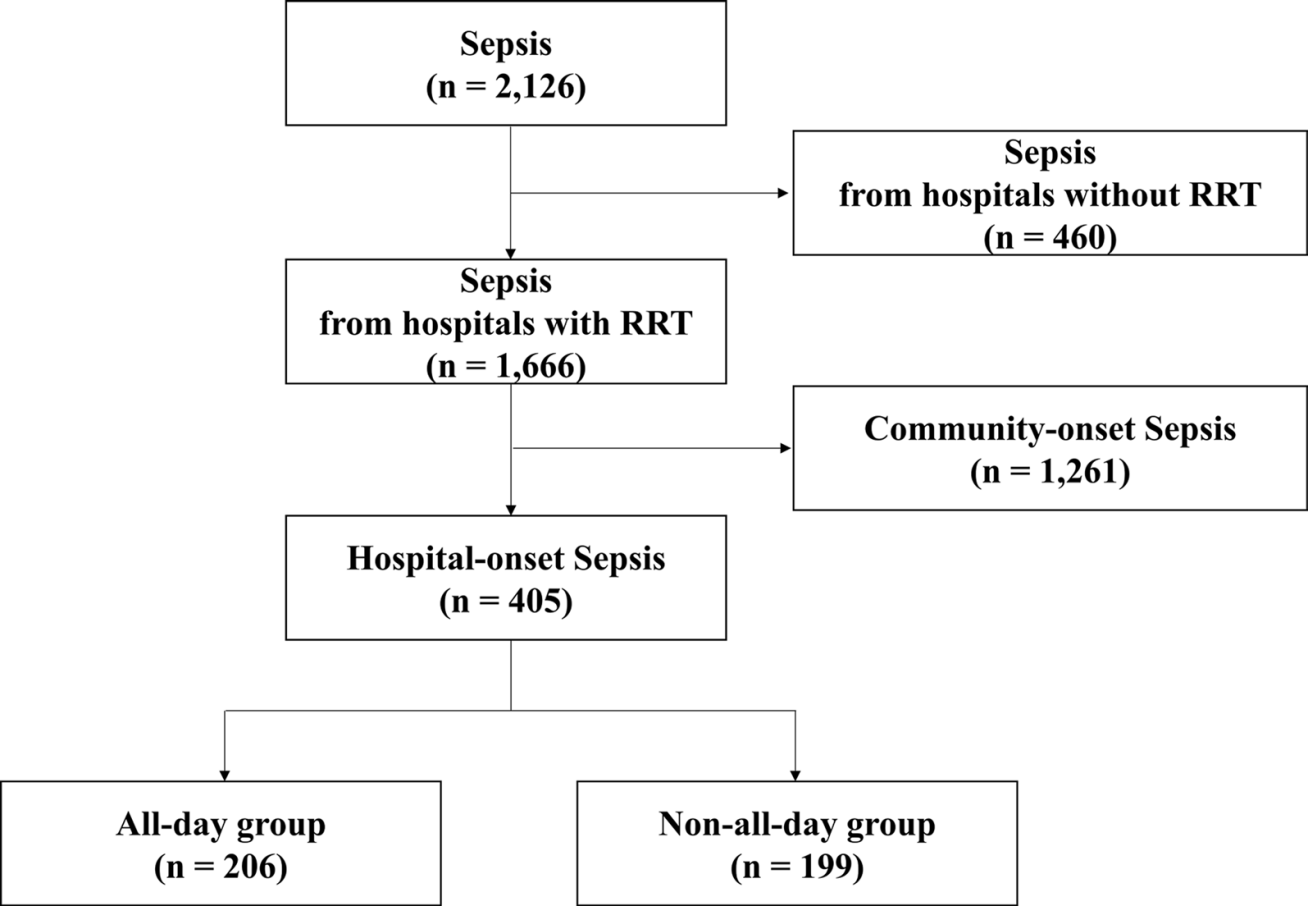
Flow diagram of the eligible study cohort from the Korea Sepsis Association Database, September 2019–February 2020**.** RRT = Rapid Response Team

Data from 2126 patients diagnosed with sepsis from 16 South Korean hospitals were recorded in the KSA database during the study period (Fig. 1). We selected 1666 patients from hospitals operating RRT. Of these, 1261 patients with community-onset sepsis were excluded. A final cohort of 405 patients met the HOS criteria. Among these 405 patients, 206 (50.9%) were admitted to hospitals operating an RRT for 24 h (all-day group), while 199 (49.1%) were admitted to hospitals that did not operate an RRT for 24 h (non-all-day group).

### Differences in characteristics

The baseline characteristics were compared between the two groups in Table 1. Patients in the all-day group were younger than those in the non-all-day group (63.2 vs. 66.3 years, *P* = 0.034). Although the CCI scores were similar, comorbidities differed between the two groups. Particularly, chronic neurologic disease was more common in the non-all-day group (10.2% vs. 19.1%, *P* = 0.011), whereas solid cancer was more frequently observed in the all-day group (51.0% vs. 40.2%, *P* = 0.030). Moreover, there were differences in the suspected site of infection. Patients in the all-day group were more likely to have an infection at a gastrointestinal site (40.3% vs. 27.6%, *P* = 0.007), but significantly more patients in the non-all-day group had a pulmonary site of infection (30.6% vs. 41.2%, *P* = 0.026). At baseline, the percentage of patients who met the criteria of septic shock in the all-day group was significantly higher than in the non-all-day group (52.4% vs. 29.1%, *P* < 0.001). Vancomycin-resistant enterococcus was more frequently found in the all-day group than that in the non-all-day group (10.2% vs. 4.5%, *P* = 0.029). There were no significant differences in sex, BMI, blood culture positivity, methicillin-resistant staphylococcus aureus, multidrug-resistant gran negative bacteria, SOFA score, or the serum lactate level between the all-day and the non-all-day groups.

**Table 1:** Characteristics of the cohort with hospital-onset sepsis according to the operating hour of the rapid response team

Variable	All-day group (*n* = 206)	Non-all-day group (*n* = 199)	*P* value
Age, year, mean ± SD	63.2 ± 14.1	66.3 ± 15.1	0.034
Sex, male, *n* (%)	140 (68.0)	122 (61.3)	0.161
BMI, kg/m^2^, mean ± SD	21.9 (3.7)	22.2 (3.9)	0.311
CCI, mean ± SD	5.4 (2.7)	5.6 (2.8)	0.582
Comorbidities, *n* (%)			
Cardiovascular disease	33 (16.0)	24 (12.1)	0.252
Chronic respiratory disease	20 (9.7)	25 (12.6)	0.361
Chronic neurologic disease	21 (10.2)	38 (19.1)	0.011
Chronic liver disease	23 (11.2)	31 (15.6)	0.192
Diabetes	62 (30.1)	58 (29.1)	0.834
Chronic renal disease	20 (9.7)	25 (12.6)	0.361
Connective tissue disease	1 (0.5)	4 (2.0)	0.209
Immunosuppressed	10 (4.9)	13 (6.5)	0.466
Hematologic malignancy	46 (22.3)	38 (19.1)	0.422
Solid cancer	105 (51.0)	80 (40.2)	0.030
Suspected site of infection, *n* (%)*			
Pulmonary	63 (30.6)	82 (41.2)	0.026
Gastrointestinal	83 (40.3)	55 (27.6)	0.007
Urinary	15 (7.3)	26 (13.1)	0.054
Skin/soft tissue	11 (5.3)	4 (2.0)	0.076
Other site**	38 (18.4)	41 (20.6)	0.584
Positive blood cultures, *n* (%)	66 (32.0)	50 (25.1)	0.124
MRSA	0 (0.0)	4 (2.0)	0.057
MDR GNB	4 (1.9)	2 (1.0)	0.685
VRE	21 (10.2)	9 (4.5)	0.029
Septic shock by Sepsis 3, *n* (%)	108 (52.4)	58 (29.1)	< 0.001
SOFA score, mean ± SD	6.6 ± 2.7	6.3 ± 3.3	0.091
Serum lactate, mmol/l, mean ± SD	4.1 ± 3.4	3.6 ± 2.8	0.145

SD = standard deviation, BMI = body mass index, CCI = Charlson comorbidity index, MDR = multidrug resistant, SOFA = sequential organ failure assessment

*13 patients—4 in the all-day group and 9 in the non-all-day group—had multiple sites of infection

**Other sites of infection include blood-stream infection, catheter-associated infection, and unknown

### Hospital outcomes

Overall, 158 of 405 patients (39.0%) died in the hospital; 73 of 206 patients (35.4%) in the all-day group, and 85 of 199 patients (42.7%) in the non-all-day group (Table 2). However, in-hospital mortality did not differ significantly between the two groups (*P* = 0.133). The hospital length of stay (median 16.0 vs. 14.0 days, *P* = 0.183) was also similar. There was no significant difference in ICU transfer between the two groups (52.4% vs. 48.2%, *P* = 0.400). Among patients admitted to the ICU, patients in the all-day group were more likely to receive mechanical ventilation (63.9% vs. 50.0%, *P* = 0.045). However, ICU mortality, ICU length of stay, continuous renal replacement therapy, and extracorporeal membrane oxygenation did not differ between the two groups.

**Table 2:** Outcomes of hospital-onset sepsis according to the operating hour of the rapid response team

Outcome measure	All-day group (*n* = 206)	Non-all-day group (*n* = 199)	*P* value
Primary outcome			
In-hospital mortality, *n* (%)	73 (35.4)	85 (42.7)	0.133
Secondary outcomes			
Hospital LOS days, median (IQR)*	16.0 (8.0–34.25)	14.0 (5.0–29.0)	0.183
Transfer to ICU, *n* (%)**	108 (52.4)	96 (48.2)	0.400
ICU mortality, *n* (%)	32 (29.6)	33 (34.4)	0.468
ICU LOS days, median (IQR)*	5.0 (2.0–11.0)	4.0 (2.0–9.0)	0.113
Mechanical ventilator, *n* (%)	69 (63.9)	48 (50.0)	0.045
CRRT, *n* (%)	32 (29.6)	41 (42.7)	0.052
ECMO, *n* (%)	2 (1.9)	0 (0.0)	0.499

LOS = length of stay, IQR = interquartile range, ICU = intensive care unit, CRRT = continuous renal replacement therapy, ECMO = extracorporeal membrane oxygenation

*Length of stay after the identification of sepsis

**The following outcomes were calculated only in patients who were transferred to the ICU

### Three-hour sepsis bundle

**Fig. 2:**
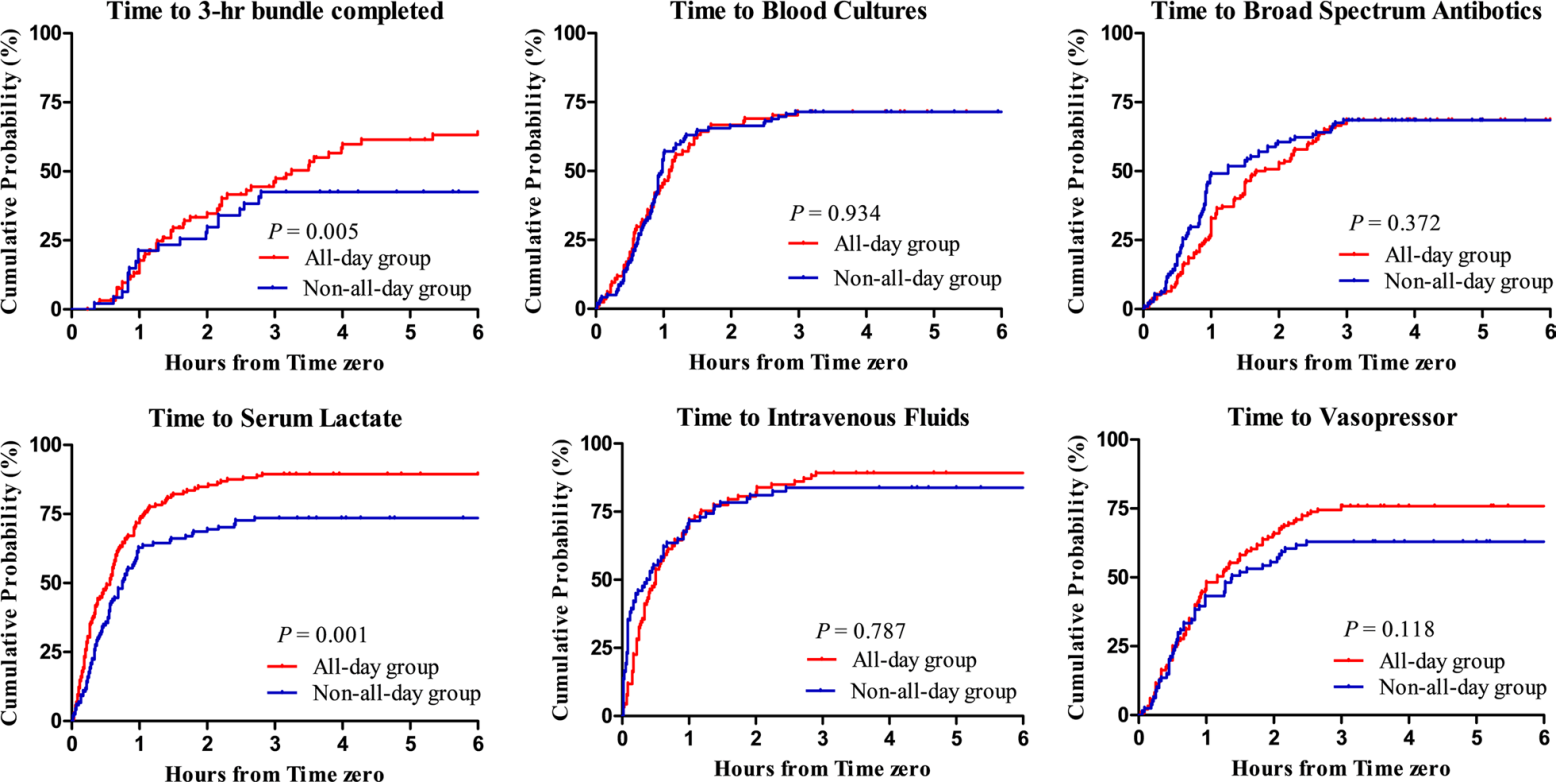
Cumulative probability of completion of the 3-h bundle and components of response team activation. Curves have been truncated at 6 h

Among 179 patients (excluding the cases in which the bundle assessment was incomplete), 100 (55.9%) had the sepsis bundle completed within 3 h (Table 3). Compared with the non-all-day group (42.6%), HOS cases in the all-day group were more likely to receive the complete sepsis bundle within 3 h (60.6%, *P* = 0.032). Serum lactate level was more frequently measured in patients in the all-day group (82.6%) than those in the non-all-day group (69.1%, *P* = 0.003). Vasopressor treatment was more often implemented in the all-day group (75.3%) than in the non-all-day group (59.6%, *P* = 0.008). Time-to-event analysis was performed for the three-hour bundle components (Fig. 2). The median time for completion of the three-hour sepsis bundle differed between the two groups (3.25 h in all-day group vs. 3.93 h in non-all-day group, *P* = 0.005). Median time to serum lactate was significantly shorter in the all-day group (0.57 h vs. 0.75 h in the non-all-day group, *P* = 0.001). Median time to blood cultures, broad-spectrum antibiotics, intravenous fluids, and vasopressors coincided between the two groups.

**Table 3:** Adherence to complete 3-h sepsis bundle in patients with hospital-onset sepsis according to the operating hour of the rapid response team

Bundle component*	All-day group	Non-all-day group	*P* value
Overall, *n* (%) (*n* = 179)	80 (60.6)	20 (42.6)	0.032
Blood cultures (*n* = 377)	70 (37.0)	86 (45.7)	0.086
Broad spectrum antibiotics (*n* = 323)	106 (57.0)	80 (58.4)	0.801
Serum lactate level testing (*n* = 350)	166 (82.6)	103 (69.1)	0.003
Intravenous fluid (*n* = 265)	143 (86.7)	81 (81.0)	0.216
Vasopressor treatment (*n* = 260)	125 (75.3)	56 (59.6)	0.008

*There were 226 incomplete bundle cases (55.8%) overall: 28 (6.9%) in blood cultures, 82 (20.2%) in broad spectrum antibiotics, 55 (13.6%) in serum lactate level testing, 140 (34.6%) in intravenous fluid, and 145 (35.8%) in vasopressor treatment

### Effects of RRT operating hours and 3-h sepsis bundle on in-hospital mortality in patients with HOS

**Fig. 3:**
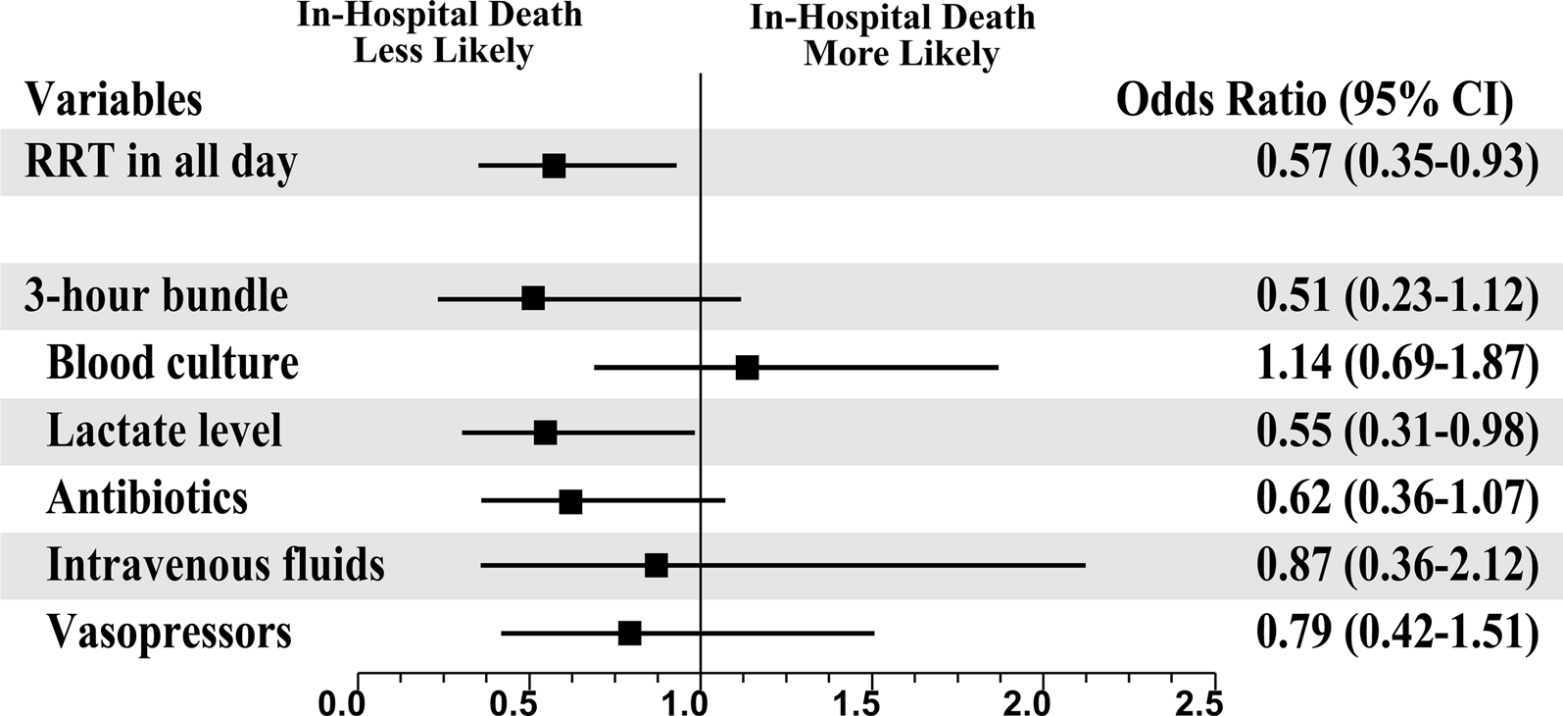
Risk-adjusted odds ratios of in-hospital death for the implementation of the rapid response team in all-day and 3-h bundle components. Here we show the odds ratios with 95% confidence intervals for in-hospital death after adjusting for the covariates. Multivariable logistic regression analysis was adjusted for selected variables based on statistic associations in the univariable analysis, including sex, body mass index, solid cancer, hematologic malignancy, gastrointestinal infection, sequential organ failure assessment score, and serum lactate level. CI = confidence interval, RRT = Rapid Response Team

The difference between the odds ratio (OR) for in-hospital mortality in the all-day group compared with the non-all-day group was not statistically significant (OR 0.74; 95% confidence interval [CI] 0.49–1.10; *P* = 0.134) (Additional file 1: Table S3). However, RRT implementation in the all-day group was associated with a significant reduction in in-hospital mortality (adjusted OR 0.57; 95% CI 0.35–0.93; *P* = 0.024) after adjustments for sex, BMI, solid cancer, hematologic malignancy, gastrointestinal infection, SOFA score, and serum lactate levels (Fig. 3). We further analyzed the association between in-hospital mortality and the completion of a three-hour bundle in patients with HOS. In an adjusted logistic regression analysis, there was no association between in-hospital mortality and the completion of the three-hour bundle (adjusted OR 0.51; 95% CI 0.23–1.12; *P* = 0.093). Similar results were observed in the association between other elements completed within 3 h and in-hospital mortality, while the completion of serum lactate testing was significantly associated with decreased in-hospital mortality (adjusted OR 0.55; 95% CI 0.31–0.98; *P* = 0.044).

## Discussion

Using a large, nationwide database from a multicenter cohort in South Korea, we found that all-day RRT was associated with a reduction in hospital mortality among patients with HOS after adjusting for confounders reflecting demographic characteristics, comorbidities, and illness severity. In our assessment of the 3-h bundle components, patients who were admitted to the hospitals operating 24-h RRTs were more likely to have a shorter time to completion of the three-hour sepsis bundle and serum lactate level measurements than those admitted to hospitals with non-all-day RRTs. However, only the completion of serum lactate testing was associated with reduced mortality in this study. Our findings support the implementation of all-day RRT for early identification and treatment of patients developing sepsis in the general ward.

Several studies have found an association between RRT and outcomes in sepsis patients in the general ward. A retrospective study comparing in-hospital mortality before and after the implementation of a hospital-wide program and RRT intervention found that the comprehensive sepsis recognition and management program was associated with a 38% reduction in death in patients diagnosed with sepsis [29]. Similarly, a previous study found that the mortality of patients with septic shock decreased from 50% before RRT initiation to 10% after RRT initiation [30]. Another study investigated the outcomes and bundle compliance of patients with septic shock in hospital wards managed through RRT [31]. This study showed that the compliance rate for achieving the sepsis bundle increased and the 28-day mortality continuously decreased over 10 years. However, no previous study has evaluated the effects of different RRT practices on the prognosis of HOS. To the best of our knowledge, this is the first attempt to investigate the association between RRT operating hours and outcomes in patients with HOS. Our study found that patients in hospitals operating all-day RRTs showed faster completion of and higher adherence to the sepsis bundle. The all-day group also showed a higher frequency of RRT activation at night, a period with low medical resources, than the non-all-day group (51.0% vs. 33.7%). Thus, screening and intervention by RRTs may help at-risk patients obtain proper and timely management. Additionally, only the completion of serum lactate testing, which is relevant for the detection of sepsis patients, was significantly associated with decreased in-hospital mortality in our study. These findings suggest that early management of HOS by all-day RRT was associated with reduced in-hospital mortality.

The criteria of HOS were defined differently depending on the study, limiting study comparability. For example, the US Centers for Disease Control and Prevention defined hospital-onset sepsis if the blood culture, first antibiotic day, and organ dysfunction all occurred on hospital day three or later [32]. To isolate inpatient processes of care, we defined patients with HOS as those who met the Sepsis-3 criteria based on the arrival time at the general ward in our study [3]. A cohort study analyzing the association of a care bundle for early sepsis management with mortality among patients with HOS used the same sepsis definition (occurring after patient arrival in an inpatient unit) as our study [8]. Another study defined HOS by time zero on an inpatient unit to compare sepsis bundle adherence in community-onset sepsis and HOS [7]. More studies must be conducted to incorporate detailed physiological, laboratory, and clinical data and provide more suitable criteria for identifying HOS.

Considering all of the incomplete bundle cases (55.8%) in our cohort, 100 (24.7%) of the 405 patients with HOS underwent the complete sepsis bundle within 3 h. We found no association between the completion of the three-hour bundle and patient outcome in this study. However, this result should be interpreted with caution since the bundle was not completed in more than half of the cases. Nevertheless, considering the adherence rates reported in other studies (13.0–30.9%), this cohort was more likely to receive proper sepsis care, and the median time of completion of the three-hour bundle in our cohort (3.58 h) was also similar to that in other cohorts (3.0 h) [7, 33]. Similar to the results of our study, a retrospective cohort study reported that completion of the sepsis bundle was not associated with improved outcomes [8]. Moreover, broad-spectrum intravenous antibiotics treatment was the only bundle component significantly associated with reduced mortality among 2296 patients with HOS. Recently, another study investigating 976 patients with sepsis in hospital wards found that the 28-day mortality was significantly associated with a complete bundle [31]. However, the only components associated with 28-day mortality among the sepsis bundle were obtaining blood cultures and lactate re-measurement. This discrepancy between the sepsis bundle components and clinical outcomes may be due to the characteristics of HOS patients with several comorbidities and high SOFA scores [3]. Moreover, other factors, such as the high adherence rate to vasopressor treatment in the all-day group, may have contributed to the lower in-hospital mortality instead of the pre-existing bundle components. Therefore, these findings suggest that the current sepsis quality metrics for HOS need refinement.

Our study has several limitations. First, we were unable to control completely for confounding from unobservable missing variables. The results may be biased, although we performed a multivariable analysis to minimize the confounding effects. A prospective, randomized study comparing the effectiveness of RRT in patients with HOS is warranted. Second, biochemical and physiological values recorded in the database were derived from the closest time before and after 24 h of the sepsis diagnosis. Thus, they may not accurately reflect events. Third, each hospital had an individual RRT structure, including composition and activation criteria. We included a large number of consecutive patients from multiple centers to increase the generalizability of these findings. Nonetheless, these differences might have resulted in a potential risk of biases. Finally, our sample may not be generalizable to patients with sepsis in other hospitals and regions. The hospitals included in this study were limited to a single nation that may have epidemiological features of sepsis distinct from those in other regions. Our findings also reflect sepsis care practices and RRT activation in South Korea.

## Conclusions

Among patients with sepsis occurring in the general ward, this nationwide study showed that all-day RRT was associated with lower in-hospital mortality compared with non-all-day RRT. We found no association between clinical outcomes and the completion of the 3-h sepsis bundle components, except for serum lactate measurements. A larger study is warranted to determine the physiologic basis of reduced mortality with all-day RRT in comparison with non-all-day RRT.

## Supplementary Information

**Additional file 1. Table S1. **Profiles of the Rapid Response Team at each center of the Korean Sepsis Alliance.** Table S2. **Rapid response team activation criteria at each center.** Table S3. **Univariable and multivariable analyses of the covariables associated with in-hospital mortality after RRT activation.

## Data Availability

The datasets used and/or analyzed during the current study are available from the corresponding author upon reasonable request.

## Abbreviations

HOS: Hospital-onset sepsis
RRT: Rapid response team
KSA: Korean Sepsis Alliance
IRB: Institutional review board
ICU: Intensive care unit
BMI: Body mass index
MDR: Multidrug resistant
SOFA: Sequential organ failure assessment score
CCI: Charlson comorbidity index
IQR: Interquartile range
OR: Odds ratio
