# Supplementary material for: Mortality of patients with hospital-onset sepsis in hospitals with all-day and non-all-day rapid response teams: a prospective nationwide multicenter cohort study
Source: Crit Care. 2022 Sep 16;26:280. doi: 10.1186/s13054-022-04149-z (PMC9482246; doi:10.1186/s13054-022-04149-z)
Supplement: Supplementary file 1 — Additional file 1. Table S1. Profiles of the Rapid Response Team at each center of the Korean Sepsis Alliance. Table S2. Rapid response team activation criteria at each center. Table S3. Univariable and multivariable analyses of the covariables associated with in-hospital mortality after RRT activation. [file 13054_2022_4149_MOESM1_ESM.docx]

**Table S1.** Profiles of the Rapid Response Team at each center of the Korean Sepsis Alliance

| **No.** | **Center** | **Operating hours per day** | **Activation frequency per day** | **No. of attending staff in operating hours** | **No. of nurses in operating hours** |
| --- | --- | --- | --- | --- | --- |
| 1 | Korea University Anam Hospital | 24 h | 0.25 | 1 | 1 |
| 2 | Seoul National University Bundang Hospital | 24 h | 10 | 2 | 2 |
| 3 | Samsung Medical Center | 24 h | 5 | 2 | 2 |
| 4 | Seoul National University Hospital | Less than 24 h, 8 h or more | 8.8 | 3 | 2 |
| 5 | Asan Medical Center | 24 h | 6 | 1 | 2 |
| 6 | Ulsan University Hospital | Less than 24 h, 8 h or more | 9 | 1 | 2 |
| 7 | Chonnam National University Hospital | 24 h | 6 | 1 | 3 |
| 8 | Jeonbuk National University Hospital | Less than 8 h | 1 | 1 | 1 |
| 9 | Chungnam National University Hospital | Less than 8 h | 1.5 | 1 | 1.5 |
| 10 | Hallym University Sacred Heart Hospital | Less than 8 h | 10 | 1 | 2 |
| 11 | Hanyang University Guri Hospital | Less than 24 h, 8 h or more | 6 | 2 | 2 |

**Table S2.** Rapid response team activation criteria at each center

| **No.** | **Center** | **Activation criteria** |
| --- | --- | --- |
| 1 | Korea University Anam Hospital | A. Screening criteria  - MEWS ≥ 2 |
| 2 | Seoul National University Bundang Hospital | - Systolic blood pressure: <90 mmHg  - Heart rate: <50, >140/min  - Respiratory rate: <10, >30/min  - Body temperature: <36°C, >39°C  - SpO_2_: <90%  - ABGA: pH < 7.25, PaCO_2_ > 50 mmHg, PaO_2_ < 55 mmHg  - Lactate > 4 mmol/L  - Total CO_2_ < 15 mmol/L |
| 3 | Samsung Medical Center | A. Calling criteria  - Respiratory: respiratory rate ≥ 30, SpO_2_ < 85% for 5 min, pH < 7.3 & PaCO_2_ > 50 mmHg, stridor, use of accessory respiratory muscles  - Cardiovascular: systolic blood pressure < 85 mmHg, heart rate > 130, acute chest pain, symptomatic arrhythmia  - Neurologic: altered mental status of sudden onset, unexplained agitation, seizure  - Others: bedside nurse concern about overall deterioration  B. Screening criteria  - MEWS ≥ 7 |
| 4 | Seoul National University Hospital | A. Calling criteria  - Respiratory: respiratory rate ≤ 8 or ≥28, SpO_2_ ≤ 90% for 5 minutes, dyspnea of sudden onset  - Cardiovascular: heart rate ≤ 40 or ≥130, systolic blood pressure ≤ 80 or ≥200, systolic blood pressure 80–90 mmHg with symptoms, chest pain not responsive to sublingual nitroglycerin  - Neurologic: altered consciousness of sudden onset, sudden paralysis of face or extremities, new onset seizure, prolonged agitation (≥10 minutes) not fully explained by medical conditions  - Others: color change of peripheral extremities, subjective judgement of attending physician or nurse  B. Screening criteria  - Heart rate: <41, >129/min  - Respiratory rate: <9, >27/min  - Systolic blood pressure: <81, >199 mmHg  - SpO_2_: <90% |
| 5 | Asan Medical Center | A. Calling criteria  - Airway: threatened, stridor  - Breathing: respiratory rate < 6 or >30/min, SpO_2_ < 90% on Venturi 40% or O_2_ 6 L/min  - Circulation: heart rate < 40 or >140/min, systolic blood pressure < 90 mmHg  - Neurology: sudden mental change, seizure  - Others: bedside nurse's concern about overall deterioration  B. Screening criteria  - Systolic blood pressure < 86 mmHg  - Sudden mental change or unexplained agitation  - Applying O_2_ > 9 L/min or FiO_2_ > 35%  - Respiratory rate > 27 or <8/min  - Unexplained heart rate > 140 or <40  - Unexplained severe metabolic acidosis: pH < 7.3, lactate > 2 mmol/L, total CO_2_ < 12 mmol/L  - PaCO_2_ > 50 mmHg or PaO_2_ ≤ 55 mmHg  - Bedside nurse concern about overall deterioration |
| 6 | Ulsan University Hospital | A. Calling criteria  - Airway: threatened airway, stridor, wheezing sound  - Breathing: respiratory rate ≤ 6 or ≥30/min, SpO_2_ < 90% on O_2_ 6 L/min or venturi mask 40%  - Circulation: heart rate < 40 or ≥140/min, systolic blood pressure < 90 mmHg  - Neurology: sudden mental change, seizure  - Others: bedside nurse’s concern about overall deterioration  B. Screening criteria  - Airway: threatened airway, stridor, wheezing sound  - Breathing: respiratory rate ≤ 6 or ≥30/min, SpO_2_ < 90%, oxygen demand (over nasal prong 6 L/min or venturi mask 40%)  - Circulation: heart rate < 40 or ≥140/min, systolic blood pressure ≤ 85 mmHg  - Neurology: sudden mental change, seizure  - Others: pH ≤ 7.33, HCO_3_ ≤ 15 mmol/L, lactate ≥ 2 mmol/L, potassium ≥ 6 mmol/L, total CO_2_ ≤ 15 mmol/L, glucose ≤ 50 mg/dL, ICU discharge patients |
| 7 | Chonnam National University Hospital | - Systolic blood pressure < 80 mmHg, heart rate < 40 or >140/min, respiratory rate ≤ 10 or ≥ 30/min, SpO_2_ ≤ 85%  - ABGA: pH < 7.3, pCO_2_ > 50 mmHg, pO_2_ < 60 mmHg  - Sudden mental change  - Symptomatic arrhythmia |
| 8 | Jeonbuk National University Hospital | A. Screening criteria  - NEWS ≥ 5 |
| 9 | Chungnam National University Hospital | A. Calling criteria  - Airway: airway obstruction sign, stridor  - Breathing: respiratory rate < 8 or >30/min, pH <7.3 & PaCO_2_ > 60 mmHg, SpO_2_ < 90% on facial mask or high-flow nasal cannula  - Circulation: heart rate <40 or >140/min, systolic blood pressure < 90 mmHg, lactate > 2 mmol/L  - Neurology: sudden mental change or unexplained agitation, seizure  - Nurse’s concern about overall deterioration  B. Screening criteria  - based on NEWS |
| 10 | Hallym University Sacred Heart Hospital | A. Screening criteria  - NEWS ≥ 7  - Respiratory rate ≥ 30/min  - Systolic blood pressure ≤ 80 mmHg  - SpO_2_ ≤ 85% |
| 11 | Hanyang University Guri Hospital | A. Calling criteria  - Airway: airway obstruction sign, stridor  - Breathing: respiratory rate < 8 or >30/min, pH < 7.3 & PaCO_2_ > 60 mmHg, SpO_2_ < 90% on facial mask or high-flow nasal cannula  - Circulation: heart rate < 40 or >140/min, systolic blood pressure < 90 mmHg, lactate > 2 mmol/L  - Neurology: sudden mental change or unexplained agitation, seizure  - Nurse’s concern about overall deterioration  B. Screening criteria  - MEWS ≥ 4  - Systolic blood pressure ≤ 80 or ≥200 mmHg, heart rate ≤ 40 or ≥130/min, respiratory rate < 9 or ≥30/min, SpO_2_ ≤ 90%  - ABGA: pH ≤ 7.3, pCO_2_ ≥ 50 mmHg, pO_2_ ≤ 60 mmHg, HCO_3_ ≤ 14 mmEq/L, lactate ≥ 4.0 mmol/L  - Electrolyte: potassium ≥ 6.0 mmol/L, total CO_2_ ≤ 14 mmol/L |

ABGA = Arterial blood gas analysis, FiO_2_ = fraction of inspired oxygen, MEWS = modified early warning score, NEWS = national early warning score, PaCO_2_ = partial pressure of carbon dioxide in arterial blood, PaO_2_ = partial pressure of oxygen in arterial blood, pCO_2_ = partial pressure of carbon dioxide, pO_2_ = partial pressure of oxygen, SpO_2_ = oxygen saturation.

| **Table S3.** Univariable and multivariable analyses of the covariables associated with in-hospital mortality after RRT activation | | | | | | | |
| --- | --- | --- | --- | --- | --- | --- | --- |
|  | Univariable analysis | | |  | Multivariable analysis | | |
| Variable | HR | 95% CI | *p-*value |  | HR | 95% CI | *p-*value |
| Age, yr | 1.01 | 0.99-1.03 | 0.108 |  |  |  |  |
| Male sex | 1.56 | 1.12-2.16 | 0.008 |  |  |  |  |
| BMI, kg/m^2^ | 1.06 | 1.00-1.11 | 0.043 |  |  |  |  |
| CCI | 1.10 | 1.02-1.19 | 0.010 |  |  |  |  |
| Comorbidities |  |  |  |  |  |  |  |
| Cardiovascular disease | 0.82 | 0.46-1.48 | 0.513 |  |  |  |  |
| Chronic respiratory disease | 1.42 | 0.76-2.66 | 0.266 |  |  |  |  |
| Chronic neurologic disease | 1.09 | 0.62-1.90 | 0.777 |  |  |  |  |
| Chronic liver disease | 1.30 | 0.73-2.31 | 0.380 |  |  |  |  |
| Diabetes | 1.01 | 0.65-1.56 | 0.967 |  |  |  |  |
| Chronic renal disease | 1.16 | 0.62-2.18 | 0.640 |  |  |  |  |
| Immunosuppressed | 0.67 | 0.27-1.67 | 0.388 |  |  |  |  |
| Hematologic malignancy | 1.56 | 0.96-2.54 | 0.070 |  |  |  |  |
| Solid cancer | 1.39 | 1.01-1.90 | 0.042 |  |  |  |  |
| Suspected site of infection |  |  |  |  |  |  |  |
| Pulmonary | 1.40 | 0.92-2.11 | 0.115 |  |  |  |  |
| Gastrointestinal | 0.44 | 0.28-0.69 | < 0.001 |  |  |  |  |
| Urinary | 1.26 | 0.64-2.50 | 0.501 |  |  |  |  |
| Skin/Soft tissue | 1.83 | 0.65-5.15 | 0.253 |  |  |  |  |
| Positive blood cultures | 0.99 | 0.64-1.54 | 0.954 |  |  |  |  |
| Septic shock by Sepsis 3 | 1.55 | 1.03-2.32 | 0.034 |  |  |  |  |
| SOFA score | 1.23 | 1.14-1.33 | < 0.001 |  |  |  |  |
| Serum lactate | 1.14 | 1.06-1.22 | 0.001 |  |  |  |  |
| 3-hour sepsis bundle completion | 0.78 | 0.41-1.39 | 0.371 |  | 0.51 | 0.23-1.12 | 0.093 |
| Blood culture | 1.10 | 0.72-1.67 | 0.667 |  | 1.14 | 0.69-1.87 | 0.617 |
| Broad spectrum antibiotics | 0.77 | 0.49-1.22 | 0.263 |  | 0.62 | 0.36-1.07 | 0.087 |
| Serum lactate level testing | 0.84 | 0.51-1.39 | 0.491 |  | 0.55 | 0.31-0.98 | 0.044 |
| Intravenous fluid | 1.10 | 0.55-2.19 | 0.785 |  | 0.87 | 0.36-2.12 | 0.762 |
| Vasopressor treatment | 1.17 | 0.68-2.01 | 0.563 |  | 0.79 | 0.42-1.51 | 0.479 |
| All-day RRT | 0.74 | 0.49-1.10 | 0.134 |  | 0.57 | 0.35-0.93 | 0.024 |

BMI = body mass index, CCI = Charlson comorbidity index, CI = confidence interval, HR = hazard ratio, RRT = rapid response team, SOFA = sequential organ failure assessment.
